# DNA Damage and Repair in Atherosclerosis: Current Insights and Future Perspectives

**DOI:** 10.3390/ijms131216929

**Published:** 2012-12-11

**Authors:** Tiziana Cervelli, Andrea Borghini, Alvaro Galli, Maria Grazia Andreassi

**Affiliations:** Institute of Clinical Physiology, CNR (The National Research Council), via Moruzzi 1, 56124 Pisa, Italy; E-Mails: andrea.borghini@ifc.cnr.it (A.B.); alvaro.galli@ifc.cnr.it (A.G.); andreassi@ifc.cnr.it (M.G.A.)

**Keywords:** DNA damage, atherosclerosis, DNA repair, miRNAs

## Abstract

Atherosclerosis is the leading cause of morbidity and mortality among Western populations. Over the past two decades, considerable evidence has supported a crucial role for DNA damage in the development and progression of atherosclerosis. These findings support the concept that the prolonged exposure to risk factors (e.g., dyslipidemia, smoking and diabetes mellitus) leading to reactive oxygen species are major stimuli for DNA damage within the plaque. Genomic instability at the cellular level can directly affect vascular function, leading to cell cycle arrest, apoptosis and premature vascular senescence. The purpose of this paper is to review current knowledge on the role of DNA damage and DNA repair systems in atherosclerosis, as well as to discuss the cellular response to DNA damage in order to shed light on possible strategies for prevention and treatment.

## 1. Introduction

**Figure 1:**
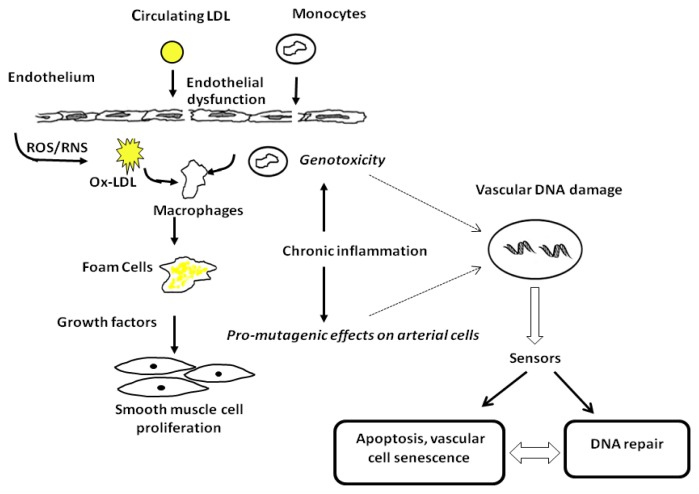
A simplified overview of atherosclerosis. The figure shows that one of the earliest events in atherosclerosis is an altered endothelial function (dysfunction), causing increased permeability to lipids, recruitment of circulating monocytes and T lymphocytes, formation of foam cells from macrophages that bind oxidatively modified LDL, secretion of inflammatory mediators and growth factors, and the accumulation of smooth muscle cells leading to the formation of an atherosclerotic plaque. In addition, the plaque environment may also induce DNA modification of vascular cells by action of endogenous DNA-damaging agents, such as reactive oxygen species (ROS) and reactive nitrogen species (RNS).

Cardiovascular disease (CVD) remains the leading cause of death in industrialized and developing countries [1]. Major clinical manifestations of CVD include myocardial infarction, coronary artery disease, stroke and peripheral artery disease [1]. In most cases, these clinical conditions result from atherosclerosis, a progressive disease of the arterial wall, characterized by focal thickening and luminal obstruction [2]. Atherosclerosis is a complex inflammatory process characterized by thickening of the intima of an artery via the accumulation of a heterogeneous mixture of cells, predominantly vascular smooth muscle cells (VSMCs), lymphocytes, macrophages and extracellular lipids, collagen and matrix. The release of growth factors and inflammatory cytokines from these various cell types promotes further accumulation of inflammatory cells and deposition of extracellular matrix components, causing the lesion to develop into an advanced plaque consisting of a lipid-rich necrotic core covered by a VSMC-rich fibrous cap. Rupture of the plaque leads to thrombosis and artery occlusion, resulting in myocardial infarction. The process is triggered by endothelial dysfunction induced by a variety of cardiovascular risk factors, such as smoking, diabetes and hypercholesterolemia, as well as genetic predisposition (Figure 1) [3,4].

Despite progress in understanding the inflammatory process of atherosclerosis, very little is known about the molecular mechanisms through which plaque is disrupted or about the relationship between plaque disruption and both the trigger and onset of acute disease. In recent years, evidence also suggests an important role for DNA damage and premature vascular aging in the development and progression of atherosclerosis [5–7]. Vascular aging is viewed as a consequence of prolonged exposure to risk factors, leading to reactive oxygen species (ROS) and reactive nitrogen species (RNS) within the plaque, which are major stimuli for DNA damage (Figure 1). Genomic instability at the cellular level can directly affect vascular function and/or lead to cell cycle arrest, apoptosis and senescence [5–7].

The purpose of this paper is to review current knowledge on the role of DNA damage and DNA repair systems in atherosclerosis and discuss the cellular response to DNA damage in order to shed light on possible strategies for prevention and treatment.

## 2. Evidence of DNA Damage in Atherosclerosis

There is increasing evidence of DNA damage accumulation in atherosclerotic plaque either as genomic alterations or as DNA adducts in nuclear DNA [8,9]. Genomic alterations observed in atherosclerotic plaque are microsatellite instability (MSI) and loss of heterozygosity (LOH). Microsatellites are DNA sequences in which a short motif of 1–5 nucleotides is tandemly repeated 10–100 times. Microsatellites are prone to mutation during replication. The loss or gain of repeated units on the daughter strand results in length variation termed microsatellite instability (MSI), and it occurs in the presence of alteration of mismatch repair (MMR). LOH is the loss of an allele that allows normal gene function, where the second allele has already been inactivated by mutation. At the vascular level, a number of studies have shown LOH and MSI in DNA extracted from atherosclerotic plaques, compared to DNA extracted from adjacent normal tissue, suggesting that specific molecular alteration of target genes could be an important molecular mechanism associated with the atherogenic process [10–13]. In addition, single or associated clonal chromosomal abnormalities were found in primary cell cultures from human atherosclerotic plaques [14]. Interestingly, Matturri *et al.* showed that unstable atherosclerotic plaques presented a variety of chromosomal abnormalities in carotid endarterectomy specimens [15]. Conversely, stable plaques did not present any chromosomal abnormalities, supporting the hypothesis that genetic instability might be of particular importance in the mechanisms of plaque evolution [15].

DNA adducts have also been detected in the VSMCs of human abdominal aorta affected by atherosclerosis, and their levels were significantly correlated with known atherogenic risk factors, including age, number of cigarettes per day currently smoked, arterial pressure, blood cholesterol and triglycerides [16]. Furthermore, these molecular end-points appear to have a critical role in disease progression and clinical outcome, as shown by findings on survival of patients affected by severe atherosclerosis after 14 years of follow-up [17]. High levels of lipid peroxidation-derived etheno DNA adducts have also been found in human atherosclerotic lesions, demonstrating the presence of DNA damage in human atherosclerotic plaque induced by both endogenous (oxidative stress and lipid peroxidation) and exogenous (environmental factors) agents [18]. It has also been shown that DNA damage not only occurs in peripheral blood cells of patients with coronary artery disease and acute myocardial infarction [19], but is also associated with the long-term clinical outcome [20].

Finally, there was also ample evidence to suggest that telomere dysfunction is another key molecular event in the pathogenesis of cardiovascular and metabolic diseases [5,6,21]. Telomeres are necessary for genomic stability and integrity, preventing chromosomal end deterioration and fusion by cellular DNA repair processes. Dysfunctional telomeres can lead to genomic instability, and telomere length is an important factor in the pathobiology of human disease [5,6]. Progressive telomere shortening has been demonstrated in peripheral blood cells from patients with metabolic syndrome, diabetes mellitus, coronary heart disease and premature myocardial infarction [5,6,21]. Importantly, accumulating data demonstrate that both telomere shortening and DNA damage are crucial mediators for vascular dysfunction by activating the DNA damage response pathway (DDR) [5,6].

In addition to the aforementioned observations that atherosclerosis is associated with the presence of DNA damage and repair thereof, evidence has emerged that DDR actually plays a causative role in vascular aging and atherosclerosis [5–7]. This evidence comes from studies in animals with an altered gene for DNA repair components, such as nucleotide excision repair (NER) proteins [22], double strand break (DSB) repair proteins [23,24], telomere maintenance proteins [25] and base excision repair (BER) proteins [26]. Before going into detail on this topic, we briefly describe the main DNA repair pathways and proteins involved, focusing on proteins that have been related to CVD.

## 3. DNA Damage Repair Overview

### 3.1. DNA Repair Systems and Key Components

In healthy cells, the variety and frequency of DNA lesions are matched by the complexity of mechanisms that counteract these threats in order to preserve genomic integrity. Collectively, these mechanisms are known as the DDR. Most DDR pathways are characterized by tightly coordinated processes: namely the detection of DNA damage, the accumulation of DNA repair factors at the site of damage and, finally, the physical repair of damage. DNA repair is strictly correlated to cell cycle progression, since the repair of DNA must occur before replication of DNA; otherwise it may result in a permanent change in the DNA sequence.

**Figure 2:**
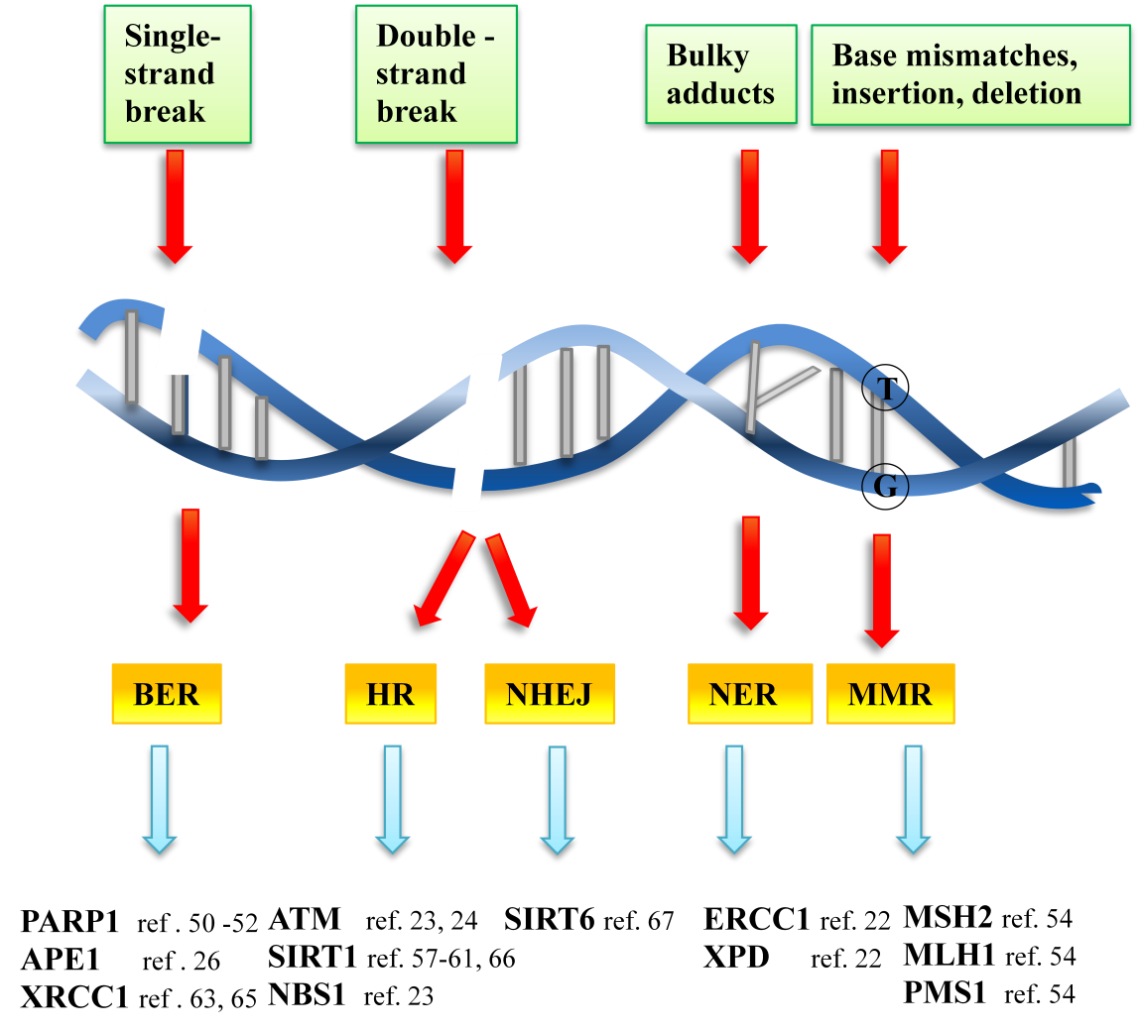
DNA damage and DDR pathways. The genome is exposed to several kinds of damage, such as SSB, DSB, bulky adducts, base mismatch, insertion and deletion. The choice of repair pathway depends on the type of lesion.

At least four main partly overlapping damage repair pathways operate in mammals: NER, BER, DSB repair, subdivided into homologous recombination (HR) and non-homologous end joining (NHEJ), and MMR pathway (Figure 2) [27]. The division of tasks among them can be roughly defined as follows. NER deals with the wide class of helix-distorting lesions that interfere with base pairing and generally obstruct transcription and normal replication. DNA surrounding the lesion is excised and then replaced using the normal DNA replication machinery. Excision repair cross-complementing protein 1 (ERCC1) is key to this excision step. XPD (also known as ERCC2) is a component of TFIIH and is a DNA helicase that is involved in 5′ to 3′ unwinding of the DNA in the vicinity of a damaged base [28]. Small chemical alterations of bases, such as oxidation, methylation, deamination and hydroxylation producing single strand breaks (SSB), are targeted by BER. These lesions may not impede transcription and replication, although they frequently miscode. In BER, damaged bases are first removed from the double helix and the ‘injured’ section of the DNA backbone is then excised and replaced with newly synthesized DNA [29]. Key to this process are the enzymes poly(ADP ribose) polymerase 1 and 2 (PARP1 and PARP2), which act as sensors and signal transducers for lesions, such as SSBs. PARP-1 detects SSBs either induced directly by ROS or generated through the enzymatic incision of an abasic site by AP endonuclease 1 (APE1) or a dual function DNA glycosylase. Upon binding to DNA damage, PARP-1 forms homodimers and catalyzes the cleavage of NAD^+^ into nicotinamide and ADP-ribose to form long branches of ADP-ribose polymers on glutamic residues of a number of target proteins. Potential targets of poly(ADP-ribosylation) include PARP-1 itself, histones, transcription factors and other signaling molecules, such as nuclear factor-kappaB, DNA-PK, p53, topoisomerase I and lamin B [30]. Poly(ADP-ribosyl)ation is involved in the regulation of multiple physiological and pathophysiological cellular functions besides DNA repair [31,32].

DSBs are the most serious lesions, as both strands are affected. Two pathways, HR and NHEJ (and presumably additional back-up systems), were developed for solving the DSB problem. The major mechanisms that cope with DSBs are HR and NHEJ. HR seems to dominate in the S and G2 phases of the cell cycle when the DNA is replicated. It is a conservative process in that it tends to restore the original DNA sequence to the site of damage [33]. Part of the DNA sequence around the DSB is removed (known as resection), and the DNA sequence on a homologous sister chromatid is used as a template for the synthesis of new DNA at the DSB site. Crucial proteins involved in mediating HR are those of MRE11 complex proteins (MRN complex, MRE11, NBS1 and RAD50). The MRN complex is highly conserved and is involved in nearly every aspect of the DSB damage response, including DNA damage sensing, signaling and repair. Other important proteins are BRCA1, BRCA2 and RAD51. DSBs are recognized by the MRE11 complex, which catalyses the activation of ataxia-telangiectasia mutated (ATM) protein [34]. In contrast to HR, NHEJ is most relevant in the G1 phase of the cell cycle, when a second copy is not available [35]. Rather than using a homologous DNA sequence to guide DNA repair, NHEJ mediates repair by directly ligating the ends of a DSB together. Sometimes, the process can cause the deletion or mutation of DNA sequences at or around the DSB site [36].

MMR is crucial to the DDR. It deals primarily with dNTP misincorporation and formation of insertion and deletion loops that form during DNA replication. These errors cause base mismatches in the DNA sequence that distort the helical structure and are recognized as DNA lesions. The recognition of the distortion triggers a sequence of events resulting in the excision of newly synthesized DNA encompassing the mismatch site and the re-synthesis of DNA in its place. Key proteins of MMR are encoded by the *mutS* and *mutL* homologue genes, such as *MSH2* and *MLH1*. A consequence of an alteration in these two proteins is MSI instability.

### 3.2. Signal Transduction Pathways of the DDR Process

DDR is a neatly regulated process employing an intricate signal transduction system. DNA lesions trigger the activation of various kinases, ATM and ATR, which constitute the primary transducers in the signaling cascade. While ATR activation is associated with SSB and stalled DNA replication forks, ATM responds mainly to DSBs. The ATM/ATR-dependent phosphorylation of H2AX at S139 constitutes one of the initial signals upon DNA lesion detection. H2AX phosphorylation is believed to be essential for the sustained accumulation of various checkpoint and DNA repair factors at DNA breaks [37]. Other ATM/ATR targets are the protein kinases CHK1 and CHK2. These proteins mediate the connection between DDR and the cell cycle. They act to reduce cyclin-dependent kinase (CDK) activity by various mechanisms, some of which are mediated by activation of the p53 transcription factor. Upon activation, p53 transcriptionally induces a host target gene, which promotes cell cycle arrest, allowing time for DNA repair [38].

Another class of signaling proteins is that of sirtuins. Sirtuins are classically known as longevity proteins. Mammalian sirtuins have diverse cellular localizations and are, therefore, involved in many cellular processes, including gene expression, metabolic control, apoptosis and cell survival, development, inflammation, neuroprotection and healthy aging [39]. SIRT1, SIRT6 and SIRT7 have nuclear localization. SIRT1 belongs to the third class of deacetylase enzymes, which require nicotinamide adenine dinucleotide (NAD^+^) as an essential cofactor. The list of SIRT1 substrates is continuously growing and includes several transcription factors—tumor suppressor protein p53 and members of the FOXO family (Forkhead box factors regulated by insulin/Akt) [40]. It has been shown that SIRT1 and SIRT6 have a role in DDR. SIRT1 has been proposed to modulate apoptosis and cell proliferation in response to damage-causing agents and metabolic imbalances [41]. One of the targets of SIRT1 is NBS1, the regulatory subunit of the MRN complex, which participates in sensing DNA damage and in mounting the cellular response to DSB [42]. SIRT6 plays a key role in DNA repair and maintenance of genomic stability in mammalian cells, integrating stress signaling to prime the DNA repair machinery in response to oxidative stress [43]. The first evidence that SIRT6 may impact on DSB repair came from the observation of genomic instability in SIRT6 knock-out mice; cells from these mice exhibit a high incidence of chromosomal rearrangement and breakage, as well as a hypersensitivity to γ-irradiation [44]. Two subsequent studies noted that knockdown of SIRT6 in human cells similarly sensitized the cells to chemically induced DSBs [45,46].

## 4. DNA Repair in Atherosclerosis

### 4.1. DNA Repair Proteins in Atherosclerosis

As announced in section 2, a causative role of DDR in the development of CVD has been proposed, according to observations in animal studies [22,23,47,48]. Durik *et al.*[22] observed functional changes in the vasculature and hypertension in two NER-defect mouse models, *Ercc1^d/−^* and *XPD^TTD^* mice. These NER defective mice showed increased vascular cell senescence, accelerated development of vasodilator dysfunction, increased vascular stiffness and elevated blood pressure at a very young age. They also found an association between SNPs of genes encoding for relevant NER components and increased vasculature stiffness and conclude that DNA repair capacity is associated with accelerated vascular aging in mice.

The activation of BER key player protein, PARP1, appears to be involved in the vascular dysfunction associated with circulatory shock, myocardial ischemia reperfusion injury, heart failure, hypertension, diabetes and cardiovascular aging [49]. Furthermore, an elevated level of PARP-1 was found in human atherosclerotic plaques [50]. Interestingly, in an ApoE mouse model of atherosclerosis fed on a high-fat diet, PARP inhibition improved endothelial function [51]. Consistent with these data, pharmacological PARP-inhibition on H_2_O_2_-induced endothelial dysfunction supports the notion that PARP inhibition may represent a potential therapeutic approach to reduce vascular dysfunction induced by oxidative stress [52].

Mahmoudi *et al.*[23] identified a mechanism by which statins accelerated DNA repair, via Hdm2 phosphorylation, NBS1 stabilization and more rapid ATM and H2AX phosphorylation. Consequently, statins attenuate DNA damage, cell senescence and telomere shortening in VSMCs and may thereby promote plaque stability in atherosclerosis [23]. Accordingly, Pernice *et al.*[53] observed a reduction in chromosomal damage in culture lymphocytes from end-stage renal disease patients with different degrees of carotid artery atherosclerosis treated with several doses of simvastatin compared to untreated cells. The finding supports the idea that the anti-atherogenic action of statins may therefore be partly ascribed to their ability to provide protection against genomic damage.

Moreover, MMR has been extensively investigated in molecular medicine. MSI is considered to indicate an ineffective MMR system. LOH and MSI have been reported in a number of human malignancies. To detect the incidence of LOH in DNA MMR genes occurring in atherosclerosis, fifty human autopsy cases of atherosclerosis were examined for LOH using 19 microsatellite markers, in three single and four tetraplex microsatellite assays. The markers used are located on or close to MMR genes. The authors found that loss of heterozygosity on hMSH2, hPMS1 and hMLH1 loci occurs in atherosclerosis. The occurrence of alterations in MMR genes indicates the presence of decreased fidelity in DNA repair in atherosclerotic tissues and may represent important events in the development of the disease [54]. MSI and LOH have been also found on chromosomes 1, 2, 8, 9 and 17 in cerebral atherosclerotic plaques [55].

### 4.2. DNA Damage Signaling Proteins in Atherosclerosis

The DDR signaling proteins have a pivotal role in atherosclerosis. Evidence of involvement of ATM in accelerated atherosclerosis has been obtained by Mercer *et al.*[24]. The authors showed that the haploinsufficiency in ATM generates defects in cell proliferation, apoptosis and mitochondrial dysfunction in VSMCs and macrophages. This failure of DNA repair in turn leads to ketosis, hyperlipidemia and increased fat storage, promoting atherosclerosis and metabolic syndrome. Moreover, it has been demonstrated that ATM is a sensor of ROS. Guo *et al.* showed that in the presence of high levels of oxidative stress, ATM is oxidized. Ox-ATM induces activation of the MRN complex, even in the absence of DNA DSBs [56].

In transgenic mice with heart-specific overexpression of SIRT1, beneficial effects are observed only in animals with a slight or moderate up-regulation of SIRT1, which is due to an increased catalase expression through FoxO-dependent signaling. Furthermore, the heart-specific overexpression of SIRT1 retards aging of the heart and further protects the heart from paraquat-induced oxidative stress [57]. Moreover, endothelial expression of SIRT1 decreases atherosclerosis in apoE null mice [58]. Polyploid endothelial cells are found in aged and atherosclerotic arteries [59,60]. Borradaile *et al.*[61] showed that human aortic endothelial cells become tetraploid as they approach replicative senescence. Interestingly, induction of polyploidy was completely prevented by modest overexpression of the NAD+ regenerating enzyme, nicotinamide phosphoribosyltransferase (Nampt). The protection from polyploidy conferred by Nampt was associated with increased SIRT1 activity, which reduced cellular ROS and the associated oxidative stress stimulus for the induction of polyploidy.

The studies presented in this section provide the best evidence to date that alteration in DDR can play a causative role in development and progression of atherosclerosis.

## 5. DNA Repair Polymorphisms and Atherosclerosis

Inter-individual variations in DNA repair capacity/efficiency linked to the presence of polymorphisms in DNA repair-related genes may account for different risks of developing vascular dysfunction and atherosclerosis. However, to date, specific studies that correlate polymorphisms or mutations in genes controlling distinct DNA repair pathways with atherosclerosis are very few [62–64]. Specifically, polymorphisms located in the DNA repair gene XRCC1, a multi-domain protein that is involved in the BER pathway and interacts with key enzymes for DNA repair, such as PARP1 and XPD, are significantly associated with the risk of coronary artery disease and elevated levels of chromosomal damage in patients [63]. An increased risk of large artery atherosclerotic stroke has been shown in a Chinese population of cigarette smokers carrying polymorphisms in several DNA repair genes [65]. In a recent study, an association between a SIRT1 gene polymorphism and cholesterol metabolism and coronary artery calcification in hemodialysis patients has been observed [66]. The authors suggest that SIRT1 polymorphism may be associated with development of cardiovascular disease in hemodialysis patients. Furthermore, Dong *et al.* show an association between the mitochondrial uncoupling proteins 5 (UCP5), SIRT6 and SIRT5 gene variants with carotid plaque, a surrogate marker of atherosclerosis [67].

A paper by Durik *et al.* also explored associations between single-nucleotide polymorphisms of selected nucleotide excision repair genes and arterial stiffness and found a significant association of a single-nucleotide polymorphism (rs2029298) in the putative promoter region of the XPE gene with carotid-femoral pulse wave velocity [22].

Finally, it is interesting to note that independent genome-wide association studies report an association between SNPs in the 9p21 locus with a risk for coronary artery disease and myocardial infarction risk [68]. This interval contains no annotated genes and is not associated with established CHD risk factors, such as plasma lipoproteins, hypertension or diabetes. On the contrary, it is located near the suppressor genes CDKN2A and CDKN2B (*cyclin-dependent kinase inhibitor 2A and 2B*). The proteins encoded by these genes p16INK4a, p15INK4b and p14ARF play a critical role in regulating cell proliferation, cell aging and the associated degeneration and the programmed cell death of many cell types. Deletion of the 9p21 locus in mice reduces CDKN2A expression and increases cultured smooth muscle cell proliferation [69], supporting the hypothesis that DNA damage and cellular senescence contribute to the pathogenesis of age-related vascular dysfunction.

## 6. miRNA, DNA Repair and Atherosclerosis

Recent studies show that miRNAs play a crucial role in the regulation of diverse cellular processes, such as proliferation, differentiation, cellular migration, apoptosis and DNA damage response [70–74]. miRNAs are an evolutionarily conserved group of small non-coding RNA molecules (18–25 nucleotides in length) that regulate the stability and translation of mRNA by perfect or imperfect base pairing at the 3′ untranslated region (3′UTR) of the mRNA [75]. miRNAs have overlapping functions and, therefore, the same miRNA may have a role in several cellular processes. Under cell stress conditions, deregulation of miRNAs is often observed and may result in the development of disease, including cardiovascular disorders, such as heart failure, cardiac hypertrophy and post-myocardial infarction remodeling [76–78]. Some endothelial-specific miRNAs play important roles in angiogenesis and are deregulated in vascular disease, such as atherosclerosis, coronary artery disease, post-angioplasty restenosis and diabetic vascular complication [79,80]. Reduced levels of circulating miR-126 in the blood of patients with coronary artery disease and diabetes have been described [81,82]. It has been shown that miR-126 is involved in angiogenesis by regulation of the activity of MAP kinase and in vascular inflammation by suppression of vascular cell adhesion molecule-1 (VCAM-1) [77]. miR-21 is abundantly expressed in all types of cardiovascular cells, such as VSMCs, endothelial cells, cardiomyocytes, cardiac fibroblast and angiogenic progenitor cells [77]. Moreover, it has been found up-regulated in human atherosclerotic plaques [83]. Thum *et al.* showed that miR-21 regulates the ERK-MAP kinase signaling pathway in cardiac fibroblasts, which impacts global cardiac structure and function [84].

The presence of DNA damage determines the activation of a cascade of proteins involved directly in the repair, but also in the control, of the cell cycle. All these steps are finely regulated. With more and more predicted and validated miRNA targets, it is becoming clear that DNA damage responsive genes have been subjected to inhibition by miRNAs and that DNA damage response results in differential activation of miRNAs [85]. Varying levels of DNA damage seemingly lead to activation of unique, as well as common sets of miRNAs, suggesting that miRNAs regulate the DNA damage response by a mechanism based on the nature and intensity of DNA damage [86]. miR-21 downregulates the expression of mismatch repair protein MSH2 [85,87], and it negatively regulates G1/S transition and G2/M checkpoint [74]. In the presence of DNA damage, miR-21 suppresses CDC25A expression through a defined sequence in 3′UTR of CDC25. A study identified that the treatment of VSMCs with hydrogen peroxide increases miR-21 expression in a dose-dependent manner and causes an anti-apoptotic effect on the cells [88]. Furthermore, several miRNAs are involved in the senescence of endothelial cells. A study indicated that miR-34a over-expression induces endothelial cell senescence and also suppresses cell proliferation by inhibiting cell cycle progression. This phenotype is determined by downregulation of SIRT1 expression and an increase of p53 acetylation [89]. Moreover, acetylated p53 increases the expression of miRNA-34a that affects SIRT1. These examples show that miRNAs target not only single mRNAs, but complete networks of often functionally related transcripts and, hence, may contribute to alter DDR in cardiovascular cells.

## 7. Conclusions and Future Perspectives

DDR is under intense investigation due to its involvement in many human diseases [90]. Alteration of DDR has a pivotal role not only in rare genetic diseases associated with mutations in DNA repair genes (e.g., Ataxia telangectasia, progerioid syndromes), but also in cancer and other degenerative diseases, such as neurodegenerative and cardiovascular diseases. In particular, DNA damage repair is an active area of investigation in the pathogenesis of atherosclerosis.

At the present time, it is well-established that atherosclerosis is in part caused by accumulated DNA damage [6]. However, our understanding of the mechanism by which DNA damage participates in the atherogenic process is still limited and fundamental questions remain unanswered. For example, can atherosclerosis result from an alteration of DDR? Can we reduce the rate of DNA damage and, thus, delay atherosclerotic progression? Challenges for the future would be to determine how the activity of DDR proteins is regulated and why DDR impacts cellular function in vascular systems. Further insight into the basic biology of DNA damage and repair is a fundamental strategy in identifying targets for novel therapeutic approaches in order to antagonize premature vascular aging. The new therapeutic strategies can be addressed in different directions. It would be useful to look for therapies aimed at potentiating DDR, as observed with statins [23], to reduce DNA damage by the antagonist of the angiotensin II receptor [7,91,92] or by the agonist of bradykinin B2 receptors [93]. Angiotensin II induces ROS-mediated DNA damage resulting in accelerated biological aging of hVSMCs via telomere-dependent and-independent pathways. Bradykinin B2 is a hormone that protects against ROS-induced DNA damage and endothelial cell senescence through bradykinin B2 receptor receptor-mediated nitric oxide (NO) release. Another strategy to reduce DNA damage could be the use of mitochondrial targeted ROS scavengers [94]. Alternatively, DDR signaling can be addressed by altering sirtuins and PARP-1 signaling, thus interfering with polyploidy and cellular senescence, correcting, in parallel, loss of proper cell function due to the oxidative stress response [49,95]. Furthermore, it would be extremely important to find molecules, like statins, improving the activity of the proteins directly involved in HR and NHEJ. On the other hand, improving our knowledge of miRNA regulation will be important for future prevention and treatment of vascular diseases, since these molecules are involved in many cellular processes, including DDR.

A better understanding of the DDR may represent the molecular substrate for explaining how environmental and occupational exposure can lead to aberrant gene expression patterns in vascular cells, resulting in increased cardiovascular risk.

*More basic research and clinical studies are needed for* a better understanding of DNA damage in vascular dysfunction in order to limit the acute complications of atherosclerosis and identify new targets for drug development.

## References

[1] Rosamond W., Flegal K., Furie K., Go A., Greenlund K., Haase N., Hailpern S.M., Ho M., Howard V., Kissela B. (2008). Heart disease and stroke statistics—2008 update: A report from the American Heart Association Statistics Committee and Stroke Statistics Subcommittee. Circulation.

[2] Ross R. (1993). Rous-Whipple Award Lecture. Atherosclerosis: A defense mechanism gone awry. Am. J. Pathol.

[3] Andreassi M.G. (2009). Metabolic syndrome, diabetes and atherosclerosis: Influence of gene-environment interaction. Mutat. Res.

[4] Libby P., Ridker P.M., Hansson G.K. (2011). Progress and challenges in translating the biology of atherosclerosis. Nature.

[5] Andreassi M.G. (2008). DNA damage, vascular senescence and atherosclerosis. J. Mol. Med. (Berl.).

[6] Wang J.C., Bennett M. (2012). Aging and atherosclerosis: mechanisms, functional consequences, and potential therapeutics for cellular senescence. Circ. Res.

[7] Herbert K.E., Mistry Y., Hastings R., Poolman T., Niklason L., Williams B. (2008). Angiotensin II-mediated oxidative DNA damage accelerates cellular senescence in cultured human vascular smooth muscle cells via telomere-dependent and independent pathways. Circ. Res.

[8] Izzotti A., Cartiglia C., Lewtas J., De Flora S. (2001). Increased DNA alterations in atherosclerotic lesions of individuals lacking the GSTM1 genotype. FASEB J.

[9] Andreassi M.G., Barale R., Iozzo P., Picano E. (2011). The association of micronucleus frequency with obesity, diabetes and cardiovascular disease. Mutagenesis.

[10] Kiaris H., Hatzistamou J., Spandidos D.A. (1996). Instability at the H-ras minisatellite in human atherosclerotic plaques. Atherosclerosis.

[11] Hatzistamou J., Kiaris H., Ergazaki M., Spandidos D.A. (1996). Loss of heterozygosity and microsatellite instability in human atherosclerotic plaques. Biochem. Biophys. Res. Commun.

[12] Spandidos D., Ergazaki M., Hatzistamou J., Kiaris H., Bouros D., Tzortzaki E., Siafakas N. (1996). Microsatellite instability in patients with chronic obstructive pulmonary disease. Oncol. Rep.

[13] McCaffrey T.A., Du B., Consigli S., Szabo P., Bray P.J., Hartner L., Weksler B.B., Sanborn T.A., Bergman G., Bush H.L. (1997). Genomic instability in the type II TGF-beta1 receptor gene in atherosclerotic and restenotic vascular cells. J. Clin. Invest..

[14] Casalone R., Granata P., Minelli E., Portentoso P., Giudici A., Righi R., Castelli P., Socrate A., Frigerio B. (1991). Cytogenetic analysis reveals clonal proliferation of smooth muscle cells in atherosclerotic plaques. Hum. Genet.

[15] Matturri L., Cazzullo A., Turconi P., Lavezzi A.M. (1997). Cytogenetic aspects of cell proliferation in atherosclerotic plaques. Cardiologia.

[16] De Flora S., Izzotti A., Walsh D., Degan P., Petrilli G.L., Lewtas J. (1997). Molecular epidemiology of atherosclerosis. FASEB J.

[17] Izzotti A., Piana A., Minniti G., Vercelli M., Perrone L., de Flora S. (2007). Survival of atherosclerotic patients as related to oxidative stress and gene polymorphisms. Mutat. Res.

[18] Nair J., de Flora S., Izzotti A., Bartsch H. (2007). Lipid peroxidation-derived etheno-DNA adducts in human atherosclerotic lesions. Mutat. Res.

[19] Demirbag R., Yilmaz R., Gur M., Kocyigit A., Celik H., Guzel S., Selek S. (2005). Lymphocyte DNA damage in patients with acute coronary syndrome and its relationship with severity of acute coronary syndrome. Mutat. Res.

[20] Federici C., Botto N., Manfredi S., Rizza A., del Fiandra M., Andreassi M.G. (2008). Relation of increased chromosomal damage to future adverse cardiac events in patients with known coronary artery disease. Am. J. Cardiol.

[21] Matthews C., Gorenne I., Scott S., Figg N., Kirkpatrick P., Ritchie A., Goddard M., Bennett M. (2006). Vascular smooth muscle cells undergo telomere-based senescence in human atherosclerosis: Effects of telomerase and oxidative stress. Circ. Res.

[22] Durik M., Kavousi M., van der Pluijm I., Isaacs A., Cheng C., Verdonk K., Loot A.E., Oeseburg H., Bhaggoe U.M., Leijten F. (2012). Nucleotide excision DNA repair is associated with age-related vascular dysfunction. Circulation.

[23] Mahmoudi M., Gorenne I., Mercer J., Figg N., Littlewood T., Bennett M. (2008). Statins use a novel Nijmegen breakage syndrome-1-dependent pathway to accelerate DNA repair in vascular smooth muscle cells. Circ. Res.

[24] Mercer J.R., Cheng K.K., Figg N., Gorenne I., Mahmoudi M., Griffin J., Vidal-Puig A., Logan A., Murphy M.P., Bennett M. (2010). DNA damage links mitochondrial dysfunction to atherosclerosis and the metabolic syndrome. Circ. Res.

[25] Perez-Rivero G., Ruiz-Torres M.P., Rivas-Elena J.V., Jerkic M., Diez-Marques M.L., Lopez-Novoa J.M., Blasco M.A., Rodriguez-Puyol D. (2006). Mice deficient in telomerase activity develop hypertension because of an excess of endothelin production. Circulation.

[26] Jeon B.H., Gupta G., Park Y.C., Qi B., Haile A., Khanday F.A., Liu Y.X., Kim J.M., Ozaki M., White A.R. (2004). Apurinic/apyrimidinic endonuclease 1 regulates endothelial NO production and vascular tone. Circ. Res.

[27] Friedberg E.C.W., Siede G.C., Wood R.D., Schultz R.A., Ellenberger T (2006). DNA Repair and Mutagenesis.

[28] Cleaver J.E., Lam E.T., Revet I. (2009). Disorders of nucleotide excision repair: The genetic and molecular basis of heterogeneity. Nat. Rev. Genet.

[29] Krokan H.E., Standal R., Slupphaug G. (1997). DNA glycosylases in the base excision repair of DNA. Biochem. J.

[30] Luo X., Kraus W.L. (2012). On PAR with PARP: Cellular stress signaling through poly(ADP-ribose) and PARP-1. Genes Dev.

[31] Kalisch T., Ame J.C., Dantzer F., Schreiber V. (2012). New readers and interpretations of poly(ADP-ribosyl)ation. Trends Biochem. Sci.

[32] Virag L., Szabo C. (2002). The therapeutic potential of poly(ADP-ribose) polymerase inhibitors. Pharmacol. Rev.

[33] Heyer W.D., Ehmsen K.T., Liu J. (2010). Regulation of homologous recombination in eukaryotes. Annu. Rev. Genet.

[34] Stracker T.H., Petrini J.H. (2011). The MRE11 complex: Starting from the ends. Nat. Rev. Mol. Cell Biol.

[35] Lieber M.R., Ma Y., Pannicke U., Schwarz K. (2003). Mechanism and regulation of human non-homologous DNA end-joining. Nat. Rev. Mol. Cell Biol.

[36] Hoeijmakers J.H. (2001). Genome maintenance mechanisms for preventing cancer. Nature.

[37] Chanoux R.A., Yin B., Urtishak K.A., Asare A., Bassing C.H., Brown E.J. (2009). ATR and H2AX cooperate in maintaining genome stability under replication stress. J. Biol. Chem.

[38] Huen M.S., Chen J. (2008). The DNA damage response pathways: At the crossroad of protein modifications. Cell Res.

[39] Pillarisetti S. (2008). A review of Sirt1 and Sirt1 modulators in cardiovascular and metabolic diseases. Recent Pat. Cardiovasc. Drug Discov.

[40] Zeng L., Chen R., Liang F., Tsuchiya H., Murai H., Nakahashi T., Iwai K., Takahashi T., Kanda T., Morimoto S. (2009). Silent information regulator, Sirtuin 1, and age-related diseases. Geriatr. Gerontol. Int.

[41] Gorospe M., de Cabo R. (2008). AsSIRTing the DNA damage response. Trends Cell Biol.

[42] Yuan Z., Seto E. (2007). A functional link between SIRT1 deacetylase and NBS1 in DNA damage response. Cell Cycle.

[43] Mao Z., Hine C., Tian X., van Meter M., Au M., Vaidya A., Seluanov A., Gorbunova V. (2011). SIRT6 promotes DNA repair under stress by activating PARP1. Science.

[44] Mostoslavsky R., Chua K.F., Lombard D.B., Pang W.W., Fischer M.R., Gellon L., Liu P., Mostoslavsky G., Franco S., Murphy M.M. (2006). Genomic instability and aging-like phenotype in the absence of mammalian SIRT6. Cell.

[45] McCord R.A., Michishita E., Hong T., Berber E., Boxer L.D., Kusumoto R., Guan S., Shi X., Gozani O., Burlingame A.L. (2009). SIRT6 stabilizes DNA-dependent protein kinase at chromatin for DNA double-strand break repair. Aging (Albany NY).

[46] Kaidi A., Weinert B.T., Choudhary C., Jackson S.P. (2010). Human SIRT6 promotes DNA end resection through CtIP deacetylation. Science.

[47] Shukla P.C., Singh K.K., Quan A., Al-Omran M., Teoh H., Lovren F., Cao L., Rovira I.I., Pan Y., Brezden-Masley C. (2011). BRCA1 is an essential regulator of heart function and survival following myocardial infarction. Nat. Commun..

[48] Okuno Y., Nakamura-Ishizu A., Otsu K., Suda T., Kubota Y. (2012). Pathological neoangiogenesis depends on oxidative stress regulation by ATM. Nat. Med.

[49] Pacher P., Szabo C. (2007). Role of poly(ADP-ribose) polymerase 1 (PARP-1) in cardiovascular diseases: the therapeutic potential of PARP inhibitors. Cardiovasc. Drug Rev.

[50] Martinet W., Knaapen M.W., De Meyer G.R., Herman A.G., Kockx M.M. (2002). Elevated levels of oxidative DNA damage and DNA repair enzymes in human atherosclerotic plaques. Circulation.

[51] Benko R., Pacher P., Vaslin A., Kollai M., Szabo C. (2004). Restoration of the endothelial function in the aortic rings of apolipoprotein E deficient mice by pharmacological inhibition of the nuclear enzyme poly(ADP-ribose) polymerase. Life Sci.

[52] Radovits T., Lin L.N., Zotkina J., Gero D., Szabo C., Karck M., Szabo G. (2007). Poly(ADP-ribose) polymerase inhibition improves endothelial dysfunction induced by reactive oxidant hydrogen peroxide *in vitro*. Eur. J. Pharmacol.

[53] Pernice F., Floccari F., Caccamo C., Belghity N., Mantuano S., Pacile M.E., Romeo A., Nostro L., Barilla A., Crasci E. (2006). Chromosomal damage and atherosclerosis. A protective effect from simvastatin. Eur. J. Pharmacol.

[54] Flouris G.A., Arvanitis D.A., Parissis J.T., Arvanitis D.L., Spandidos D.A. (2000). Loss of heterozygosity in DNA mismatch repair genes in human atherosclerotic plaques. Mol. Cell Biol. Res. Commun.

[55] Miniati P., Sourvinos G., Michalodimitrakis M., Spandidos D.A. (2001). Loss of heterozygosity on chromosomes 1, 2, 8, 9 and 17 in cerebral atherosclerotic plaques. Int. J. Biol. Markers.

[56] Guo Z., Kozlov S., Lavin M.F., Person M.D., Paull T.T. (2010). ATM activation by oxidative stress. Science.

[57] Alcendor R.R., Gao S., Zhai P., Zablocki D., Holle E., Yu X., Tian B., Wagner T., Vatner S.F., Sadoshima J. (2007). Sirt1 regulates aging and resistance to oxidative stress in the heart. Circ. Res.

[58] Zhang Q.J., Wang Z., Chen H.Z., Zhou S., Zheng W., Liu G., Wei Y.S., Cai H., Liu D.P., Liang C.C. (2008). Endothelium-specific overexpression of class III deacetylase SIRT1 decreases atherosclerosis in apolipoprotein E-deficient mice. Cardiovasc. Res.

[59] Tokunaga O., Fan J.L., Watanabe T. (1989). Atherosclerosis- and age-related multinucleated variant endothelial cells in primary culture from human aorta. Am. J. Pathol.

[60] Aviv H., Khan M.Y., Skurnick J., Okuda K., Kimura M., Gardner J., Priolo L., Aviv A. (2001). Age dependent aneuploidy and telomere length of the human vascular endothelium. Atherosclerosis.

[61] Borradaile N.M., Pickering J.G. (2010). Polyploidy impairs human aortic endothelial cell function and is prevented by nicotinamide phosphoribosyltransferase. Am. J. Physiol. Cell Physiol.

[62] Bazo A.P., Salvadori D., Salvadori R.A., Sodre L.P., da Silva G.N., de Camargo E.A., Ribeiro L.R., Salvadori D.M. (2011). DNA repair gene polymorphism is associated with the genetic basis of atherosclerotic coronary artery disease. Cardiovasc. Pathol..

[63] Guven M., Guven G.S., Oz E., Ozaydin A., Batar B., Ulutin T., Hacihanefioglu S., Domanic N. (2007). DNA repair gene XRCC1 and XPD polymorphisms and their association with coronary artery disease risks and micronucleus frequency. Heart Vessels.

[64] Mahmoudi M., Mercer J., Bennett M. (2006). DNA damage and repair in atherosclerosis. Cardiovasc. Res.

[65] Shyu H.Y., Shieh J.C., Ji-Ho L., Wang H.W., Cheng C.W. (2012). Polymorphisms of DNA repair pathway genes and cigarette smoking in relation to susceptibility to large artery atherosclerotic stroke among ethnic Chinese in Taiwan. J. Atheroscler. Thromb.

[66] Shimoyama Y., Mitsuda Y., Tsuruta Y., Suzuki K., Hamajima N., Niwa T. (2012). SIRTUIN 1 gene polymorphisms are associated with cholesterol metabolism and coronary artery calcification in Japanese hemodialysis patients. J. Ren. Nutr.

[67] Dong C., Della-Morte D., Wang L., Cabral D., Beecham A., McClendon M.S., Luca C.C., Blanton S.H., Sacco R.L., Rundek T. (2011). Association of the sirtuin and mitochondrial uncoupling protein genes with carotid plaque. PLoS One.

[68] Samani N.J., Schunkert H. (2008). Chromosome 9p21 and cardiovascular disease: the story unfolds. Circ. Cardiovasc. Genet.

[69] Visel A., Zhu Y., May D., Afzal V., Gong E., Attanasio C., Blow M.J., Cohen J.C., Rubin E.M., Pennacchio L.A. (2010). Targeted deletion of the 9p21 non-coding coronary artery disease risk interval in mice. Nature.

[70] Crippa S., Cassano M., Sampaolesi M. (2012). Role of miRNAs in muscle stem cell biology: Proliferation, differentiation and death. Curr. Pharm. Des.

[71] Karp X., Ambros V. (2005). Developmental biology. Encountering microRNAs in cell fate signaling. Science.

[72] Miska E.A., Alvarez-Saavedra E., Townsend M., Yoshii A., Sestan N., Rakic P., Constantine-Paton M., Horvitz H.R. (2004). Microarray analysis of microRNA expression in the developing mammalian brain. Genome Biol..

[73] Xu P., Guo M., Hay B.A. (2004). MicroRNAs and the regulation of cell death. Trends Genet.

[74] Hu H., Gatti R.A. (2011). MicroRNAs: New players in the DNA damage response. J. Mol. Cell Biol.

[75] Bartel D.P. (2009). MicroRNAs: Target recognition and regulatory functions. Cell.

[76] Ikeda S., Kong S.W., Lu J., Bisping E., Zhang H., Allen P.D., Golub T.R., Pieske B., Pu W.T. (2007). Altered microRNA expression in human heart disease. Physiol. Genomics.

[77] Hartmann D., Thum T. (2011). MicroRNAs and vascular (dys)function. Vascul. Pharmacol.

[78] Thum T. (2012). MicroRNA therapeutics in cardiovascular medicine. EMBO Mol. Med.

[79] Hammond S.M. (2006). RNAi, microRNAs, and human disease. Cancer Chemother. Pharmacol.

[80] Mann D.L. (2007). MicroRNAs and the failing heart. N. Eng. J. Med.

[81] Fichtlscherer S., De Rosa S., Fox H., Schwietz T., Fischer A., Liebetrau C., Weber M., Hamm C.W., Roxe T., Muller-Ardogan M. (2010). Circulating microRNAs in patients with coronary artery disease. Circ. Res.

[82] Zampetaki A., Kiechl S., Drozdov I., Willeit P., Mayr U., Prokopi M., Mayr A., Weger S., Oberhollenzer F., Bonora E. (2010). Plasma microRNA profiling reveals loss of endothelial miR-126 and other microRNAs in type 2 diabetes. Circ. Res.

[83] Raitoharju E., Lyytikainen L.P., Levula M., Oksala N., Mennander A., Tarkka M., Klopp N., Illig T., Kahonen M., Karhunen P.J. (2011). miR-21, miR-210, miR-34a, and miR-146a/b are up-regulated in human atherosclerotic plaques in the Tampere Vascular Study. Atherosclerosis.

[84] Thum T., Gross C., Fiedler J., Fischer T., Kissler S., Bussen M., Galuppo P., Just S., Rottbauer W., Frantz S. (2008). MicroRNA-21 contributes to myocardial disease by stimulating MAP kinase signalling in fibroblasts. Nature.

[85] Wan G., Mathur R., Hu X., Zhang X., Lu X. (2011). miRNA response to DNA damage. Trends Biochem. Sci.

[86] Simone N.L., Soule B.P., Ly D., Saleh A.D., Savage J.E., Degraff W., Cook J., Harris C.C., Gius D., Mitchell J.B. (2009). Ionizing radiation-induced oxidative stress alters miRNA expression. PLoS One.

[87] Yu Y., Wang Y., Ren X., Tsuyada A., Li A., Liu L.J., Wang S.E. (2010). Context-dependent bidirectional regulation of the MutS homolog 2 by transforming growth factor beta contributes to chemoresistance in breast cancer cells. Mol. Cancer Res.

[88] Lin Y., Liu X., Cheng Y., Yang J., Huo Y., Zhang C. (2009). Involvement of MicroRNAs in hydrogen peroxide-mediated gene regulation and cellular injury response in vascular smooth muscle cells. J. Biol. Chem.

[89] Ito T., Yagi S., Yamakuchi M. (2010). MicroRNA-34a regulation of endothelial senescence. Biochem. Biophys. Res. Commun.

[90] Jackson S.P., Bartek J. (2009). The DNA-damage response in human biology and disease. Nature.

[91] Kobayashi K., Imanishi T., Akasaka T. (2006). Endothelial progenitor cell differentiation and senescence in an angiotensin II-infusion rat model. Hypertens. Res.

[92] Imanishi T., Kobayashi K., Kuroi A., Mochizuki S., Goto M., Yoshida K., Akasaka T. (2006). Effects of angiotensin II on NO bioavailability evaluated using a catheter-type NO sensor. Hypertension.

[93] Oeseburg H., Iusuf D., van der Harst P., van Gilst W.H., Henning R.H., Roks A.J. (2009). Bradykinin protects against oxidative stress-induced endothelial cell senescence. Hypertension.

[94] Addabbo F., Montagnani M., Goligorsky M.S. (2009). Mitochondria and reactive oxygen species. Hypertension.

[95] Carafa V., Nebbioso A., Altucci L (2012). Sirtuins and disease: The road ahead. Front. Pharmacol..

